# Uncovering the antifungal potential of Cannabidiol and Cannabidivarin

**DOI:** 10.1371/journal.pntd.0013081

**Published:** 2025-06-05

**Authors:** Hue Dinh, Kenya E. Fernandes, Paige E. Erpf, Evie J. M. Clay, Aidan P. Tay, Stephanie S. Nagy, Sebastian Schaefer, Ram Maharjan, Megan D. Lenardon, Dylan H. Multari, Paul A. Haynes, Ian T. Paulsen, Marina J. Santiago, Mark Connor, Dee Carter, Amy K. Cain

**Affiliations:** 1 School of Natural Sciences, Macquarie University, North Ryde, New South Wales, Australia; 2 ARC Centre of Excellence in Synthetic Biology, School of Natural Sciences, Macquarie University, North Ryde, New South Wales, Australia; 3 School of Life and Environmental Sciences, The University of Sydney, Sydney, New South Wales, Australia; 4 Sydney Institute for Infectious Diseases, The University of Sydney, Sydney, New South Wales, Australia; 5 School of Biological Sciences, University of Bristol, Bristol, United Kingdom; 6 School of Biotechnology and Biomolecular Sciences, UNSW, Sydney, New South Wales, Australia; 7 School of Chemical Engineering, UNSW, Sydney, New South Wales, Australia; 8 Australian Research Council, Industrial Transformation Training Centre for Facilitated Advancement of Australia’s Bioactives (FAAB), Macquarie University, Sydney, New South Wales, Australia; 9 Macquarie Medical School, Macquarie University, North Ryde, New South Wales, Australia; University of Florida, UNITED STATES OF AMERICA

## Abstract

Fungal infections pose a major threat to human health with increasing incidence of antifungal resistance globally. Despite the need for novel antifungal drugs, few are currently in clinical development. Here we evaluate the antifungal activity of five phytocannabinoids against several clinically relevant fungal pathogens, with a focus on the priority pathogen *Cryptococcus neoformans*. Our results demonstrate that Cannabidiol (CBD), and particularly Cannabidivarin (CBDV), have broad activity against *C. neoformans* and other fungal pathogens, including dermatophytes that cause common tinea. We found that both CBD and CBDV acted in a fungicidal manner and prevented biofilm formation in *C. neoformans*. Phytocannabinoid treatment impeded factors important for virulence and antifungal resistance, including reduced capsule size and disruption of mature biofilms. Proteomics analysis revealed that the antifungal activity of CBD and CBDV was linked to destabilisation of the membrane, alterations in ergosterol biosynthesis, disruption of metabolic pathways, as well as selective involvement of mitochondrial-associated proteins. We next tested the ability of CBD to topically clear a *C. neoformans* fungal infection in vivo using the *Galleria mellonella* burn wound model, and we observed greatly improved survival in the CBD treated larvae. This study illustrates the potential of phytocannabinoids as antifungal treatments and opens up new routes towards development of novel antifungal drugs.

## Author summary

Fungal infections are a major public health issue affecting over a billion people globally. Current antifungal treatments are increasingly compromised by drug resistance and show adverse side effects, underscoring the urgent need for novel therapies. Phytocannabinoids like Cannabidiol (CBD) and Cannabidivarin (CBDV), which have established safety profiles and are approved or under investigation for neurological conditions, may hold promise in this domain. Despite this, their antifungal properties remain underexplored. Here we show that CBD and CBDV exhibit *in vitro* antifungal activity against various fungi, including common dermatophytes causing “jock itch” and “athlete’s foot”, as well as WHO Critical Priority pathogens, such as *Cryptococcus neoformans*. Further investigation in *C. neoformans* revealed that CBD and CBDV appear to work by disrupting biofilms, altering fungal cell morphology, and impacting metabolic pathways and membrane integrity, as observed through comparative proteomics. Further, *in vivo* experiments using *Galleria mellonella* infected with *C. neoformans* revealed significantly improved survival with CBD treatment. The *in vitro* and *in vivo* antifungal efficacy of CBD and CBDV established in this study highlights the potential of phytocannabinoids to address the pressing need for effective and new treatments for fungal infections.

## Introduction

Fungal infections are a growing public health concern, killing more than 3.8 million people, and affecting over a billion, per year globally [1–4]. The substantial impact that fungal pathogens have on human health has gained recent notoriety, with the World Health Organization (WHO) publishing the first fungal priority pathogens list in late 2022. Four organisms were listed in the WHO’s top category “Critical Priority Group” based on their high disease burden and resistance to antifungals: *Cryptococcus neoformans, Candida auris, Aspergillus fumigatus*, and *Candida albicans* [5]. Other fungal pathogens not on this list also pose significant challenges to public health including, dermatophytes which cause tinea and are increasing in prevalence [6], along with *Rhizopus oryzae, Mucor circinelloides,* and *Fusarium oxysporum* complexes, which contribute to the complexity of fungal infections and cause hard-to-treat conditions such as onychomycosis, mucormycosis, and zygomycosis, respectively [7–9]. In this study, we assessed the potential of phytocannabinoids as an antifungal agent to tackle critical priority and common fungal pathogens, focussing on *C. neoformans* as as target for new therapies.

*C. neoformans* is an encapsulated yeast-like environmental fungus that is found on all continents except Antarctica [10]. Human exposure occurs frequently through the inhalation of fungal spores or desiccated yeast cells [11]. While normally self-limiting and asymptomatic, infection can lead to pneumonia and can progress to life-threatening meningitis and meningoencephalitis, particularly in HIV-AIDS patients, solid organ transplant recipients, cancer patients, intravenous drug users, and individuals with conditions requiring immunosuppressive drug therapy [12–16]. Members of the closely related *Cryptococcus gattii* species complex can infect otherwise healthy people and are considered primary pathogens.

There are currently five common classes of antifungal drugs available for superficial and systemic antifungal therapies including azoles and allylamines that target the ergosterol biosynthetic pathway, polyenes that target ergosterol directly, echinocandins that inhibit cell wall β-glucan synthesis, and pyrimidine analogues that block protein synthesis or inhibit DNA replication [17,18]. The treatment of cryptococcosis infection typically involves induction with the polyene, amphotericin B (AmpB), which is frequently paired with the pyrimidine analog, 5-flucytosine (5-FC), and followed by maintenance therapy with an azole drug, most commonly fluconazole (FLC) [19]. However, treatment can present unwanted issues, including severe nephrotoxicity associated with prolonged administration of AmpB, myelosuppression, and gastrointestinal disturbance associated with 5-FC treatment, and frequent induction of resistance to FLC [20,21]. Unfortunately, despite the rising demand for new drugs there are very few antifungals in the development pipeline. Thus, we are clearly in urgent need of novel, accessible and effective therapies for fungal infections, particularly cryptococcosis.

**Fig 1:**
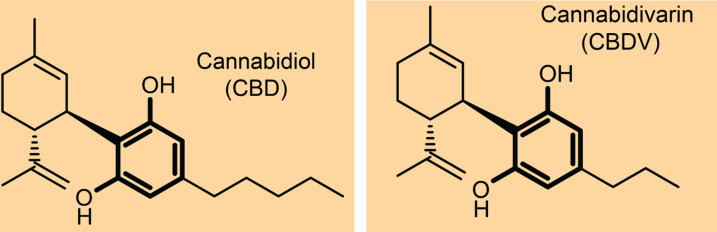
Chemical structure of CBD and CBDV.

Phytocannabinoids are bioactive natural products found in some flowering plants, liverworts, and fungi [22]. Hundreds of phytocannabinoids have been isolated from *Cannabis sativa* Linnaeus [23], a plant with a rich history of medicinal use dating back to ancient times [24]. The non-psychoactive phytocannabinoids in *C. sativa* with bioactivity in humans include Cannabidiol (CBD) and Cannabidivarin (CBDV; chemical structures shown in **Fig 1**). CBD is among the most abundant phytocannabinoids occurring in the cannabis plant, whilst CBDV is less common [25]. Currently, CBD is approved for treatment in a range of conditions including chronic pain, bladder function, nausea, and intractable epilepsy in several countries, while CBDV is undergoing clinical trials for its treatment of epilepsy and other neurological conditions unrelated to infectious disease. One attractive feature of expanding phytocannabinoid therapy as an antifungal is the favourable safety profile in humans; for example CBD has had minimal side effects reported and may be tolerated by patients unable to take traditional antifungals, such as those allergic [26]. Further, chronic use (up to 26 days) and high doses of CBD (up to 1,500 mg/day) are reportedly well tolerated in humans [27].

A handful of previous studies have reported antifungal activity of cannabis extracts containing complex mixture of natural cannabinoids against different fungus, namely *C. albicans*, *Candida krusei* (now *Pichia kudriavzevii*)*, Saccharomyces cerevisiae*, and *C. neoformans* [28–30]. However, very little work has been carried out to assess the broader activity of purified diverse phytocannabinoids against diverse fungal species, including *Cryptococcus* spp. One study showed antifungal activity of CBDV against *C. albicans* at 3.4 µg/mL [31] while a recent larger antimicrobial screen identified some extremely low-level antifungal effects of CBD (at 128 μg/ml) on *C. neoforman*s, but not *C. albicans* [32]. Further, little is known about the underlying mechanisms of antifungal action for CBD and its derivatives, with one mechanistic study observing that CBD prevents biofilm formation and dispels mature biofilms in *C. albicans* [33]. The polysaccharide capsule surrounding *C. neoformans* is essential for biofilm formation [34], and thus abundance of capsule-related proteins may provide insights into the antibiofilm effect of phytocannabinoids.

This study aimed to: (i) assess the antifungal activity of bioactive phytocannabinoids, cannabidiol (CBD) and cannabidivarin (CBDV) against *Cryptococcus neoformans*; (ii) investigate their antifungal activity across a broader spectrum of *Cryptococcus* species and other clinically significant pathogenic fungi, including *Aspergillus* spp., dermatophytes, and hard-to-treat moulds such as *Mucor circinelloides*, *Rhizopus oryzae*, and *Fusarium oxysporum* complex; and (iii) explore the underlying mechanisms of action, including the effects of phytocannabinoids on capsule production in *C. neoformans*.

## Results and discussion

### CBD and CBDV are active against *C. neoformans*

Two phytocannabinoids, CBD and CBDV, were screened against model strain *C. neoformans var. grubii* H99 ATCC 208821 (termed H99 hereafter) in accordance with the Clinical and Laboratory Standards Institute (CLSI) guidelines for fungal susceptibility testing. Following these CLSI protocols that specify use of RPMI-1640 (pH 7.4) as the test medium, we found that CBD and CBDV exhibited antifungal activity against *C. neoformans* H99 with minimum inhibitory concentrations (MIC) of 6.25 μg/mL and 12.5 μg/mL, respectively (**Table 1**). We tested the MICs of phytocannabinoids under different conditions and found that the MIC value varies depending on pH and media selection. Specifically, both RPMI-1640 (pH = 4.0) and YNB (pH = 4.5) increased the MIC of CBD and CBDV by 2–4-fold, as compared to RPMI (pH = 7.4) (**Table 1**). In fact, the use of YNB as the test medium in a previous study may explain the lack of antifungal activity that they observed and may represent underreporting of antifungal activity more generally [32].

**Table 1:** Minimum inhibitory concentration (MIC) and minimum fungicidal concentration (MFC) of phytocannabinoids and currently used antifungals (μg/mL) against *C. neoformans* H99.

Agent	RPMI-1640				Yeast Nitrogen Base* (YNB)
	pH 7.4		pH 4.0		pH 4.5
	MIC	MFC	MIC	MFC	MIC
AmpB	0.45	1	0.5	1	1
CBD	6.25	25	25	>25	>25
CBDV	12.5	50	25	>25	12.5

**Fig 2:**
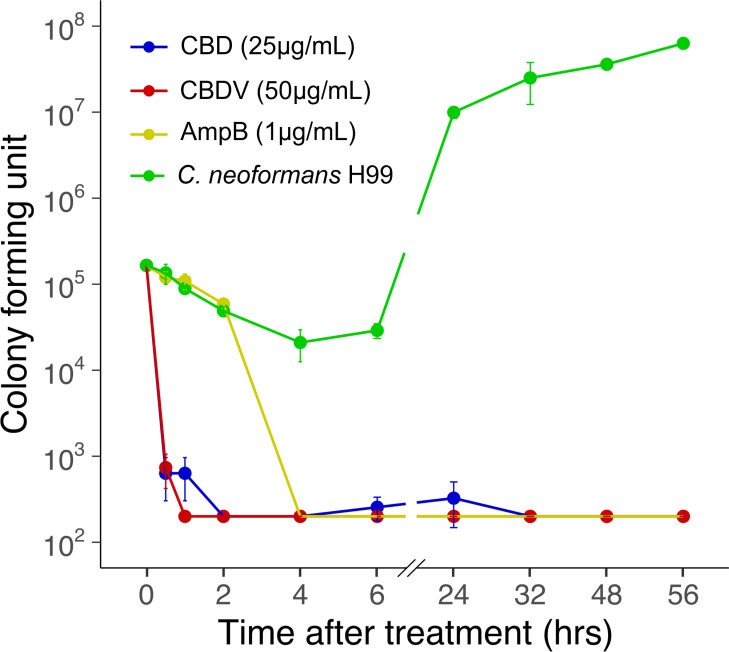
Phytocannabinoids exhibit fungicidal activity against *C. neoformans* H99. Time-kill assay of *C. neoformans* H99 cells treated with CBD (shown in blue), CBDV (red), and AmpB (yellow) at 2 × MIC compared to an untreated control (green) over 56 hr. Data are mean ± SD for *n* = 2 replicates.

Next, we tested whether CBD and CBDV acted on *C. neoformans* H99 in fungicidal or fungistatic manner. Minimum fungicidal concentrations (MFC) were determined by back plating of fungal cells treated with different concentrations of CBD, CBDV, or AmpB, until no visible growth was observed. The results showed that CBD, CBDV, and AmpB are fungicidal at 25 μg/mL, 50 μg/mL, and 1 μg/mL, respectively (S1 Fig). This concentration was then used for a time-kill assay to understand time-dependent effect of CBD and CBDV on *C. neoformans* H99. The result showed a rapid killing effect of both CBD and CBDV at 30 min after treatment, which was considerately faster than the AmpB control where killing was observed after 4 hr (Fig 2).

Next, we investigated whether phytocannabinoids interact with currently used antifungals such as AmpB and fluconazole by performing fractional inhibitory concentration (FIC) assays. The drug pairing results showed no synergistic or antagonistic effects observed between phytocannabinoids and standard antifungals including AmpB and fluconazole (S2 Fig), thus the phytocannabinoids do not seem to interact with existing antifungals.

### CBD and CBDV exhibit activity against specific fungal pathogens from diverse sources

We sought to determine whether effect of CBD and CBDV is specific to *C. neoformans* H99 or active against broad range of other fungal pathogens. For this purpose, we tested 33 fungal strains including WHO critically important pathogens (*C. albicans*, *C. auris*, and *A. fumigatus*) and diverse strains from a range of sources (veterinary, clinical, and environment; **Table 2**).

**Table 2:** Antifungal activity of phytocannabinoids on diverse fungal species.

Species	Strain	Country	Source	MIC (μg/ml)^1^		
				CBD	CBDV	AmpB
***Cryptococcus***						
*C. neoformans* (VNI)	CH40–01 ^2^	Bangkok	Clinical	>25	12.5	0.5
*C. neoformans* (VNII)	571 114 ^2^	Australia	Veterinary	>25	12.5	–
	WM626 ^2^	Australia	Clinical	>25	12.5	–
*C. neoformans* (VNIII)	WM628 ^2^	Australia	Clinical	>25	12.5	0.25
*Cryptococcus deneoformans* (VNIV)	WM629 ^2^	Australia	Clinical	3.13	12.5	–
*Cryptococcus gattii* (VGI)	2005/215 ^2^	France	Clinical	>25	12.5	0.25
*Cryptococcus deuterogattii* (VGII)	R265 ^2^	French Guyana	Clinical	>25	12.5	0.5
*Cryptococcus bacillisporus* (VGIII)	97/427 ^2^	Mexico	Clinical	>25	12.5	0.5
*Cryptococcus tetragattii* (VGIV)	MMRL3013 ^2^	Botswana	Clinical	>25	25	0.25
***Candida***						
*C. albicans*	SC5314 ^2^	Australia	Clinical	>25	12.5	0.25
	03-266-3110 ^2^	Australia	Clinical	>25	>25	–
*C. auris*	CAU-05	Venezuela	Clinical	>25	>25	–
	CAU-09	India	Clinical	>25	>25	–
*Candida dubliniensis*	AMMRL1881 ^2^	Australia	Clinical	>25	12.5	0.5
*Candida glabrata*	M74	Australia	Clinical	>25	>25	–
*Candida parapsilosis*	ATCC22018 ^2^	Australia	Clinical	>25	>25	0.5
*Candida tropicalis*	M230640 ^2^	Australia	Clinical	>25	>25	1
*Clavispora lusitaniae (Candida lusitaniae)*	M2002 ^2^	Australia	Clinical	>25	>25	0.5
*Kluyveromyces marxianus (Candida kefyr)*	M1896 ^2^	Australia	Clinical	>25	>25	1
*Meyerozyma guilliermondii (Candida guilliermondii)*	AMMRL1830 ^2^	Australia	Clinical	>25	25	0.25
*Pichia kudriavzevii (Candida krusei)*	ATCC6248	Australia	Clinical	>25	>25	0.5
***Aspergillus***						
*Aspergillus flavus*	ATCC204304	Australia	Clinical	>25	>25	0.5
*A. fumigatus*	ATCC204305	Australia	Clinical	>25	>25	0.5
*Aspergillus lentulus*	DTO 341-G7	Australia	Veterinary	>25	>25	1
*Aspergillus niger*	5181^3^	Australia	Environmental	>25	>25	1
*Aspergillus terreus* cpx	75-16-089-2500^4^	Australia	Clinical	>25	>25	1
**Dermatophytes**						
*Microsporum canis*	R236	Australia	Clinical	1.56	6.25	0.5
*Nannizzia gypsea (Microsporum gypseum)*	R133	Australia	Clinical	3.13	6.25	0.5
*Trichophyton interdigitale*	CTI1	Australia	Clinical	1.56	6.25	0.5
*Trichophyton rubrum*	CTR1	Australia	Clinical	3.13	6.25	0.5
**Other Moulds**						
*M. circinelloides*	80-14-240-5007^5^	Australia	Clinical	6.25	>25	–
*R. oryzae*	MTC04	Australia	Environmental	>25	12.5	–
*F. oxysporum* cpx	MTC05	Australia	Environmental	>25	>25	–

^1^ MIC values that could be determined in this assay (≤25 µg/ml) are coloured in blue and those above in red. AmpB MICs were sourced from previously collected data, where available and included for comparison.

2 3 4 5 Strains first published in [47–50], respectively.

We found that, within the genus *Cryptococcus,* CBDV was consistently active against all nine strains tested, spanning six species, each with a MIC of 12.5 μg/mL. Although CBD was only active against one of the nine *Cryptococcus* strains (*C. deneoformans* (VNIV)), it displayed a lower MIC of 3.13 µg/mL (Table 2**).** These results indicate that phytocannabinoids could be promising potential leads for not only the treatment of immunosuppressed patients caused by *C. neoformans* but also community cases caused by other strains of *Cryptococcus* such as *C. gattii* complex, which occurs predominantly in healthy patients [35–37].

Among *Candida* species tested, CBDV was active against only three of the 12 strains including *C. albicans, C. dubliniensis* (MIC = 12.5 µg/mL), and *C. guilliermondii* (MIC = 25 µg/mL), while CBD was not active against any strains up to the highest concentration tested (25 µg/mL). This indicates that phytocannabinoids are not as broadly active across *Candida* spp. and may not be appropriate as broad-spectrum antifungals.

Encouragingly, both CBD and CBDV showed relatively potent antifungal activity against all tinea-causing dermatophytes, with MICs ranging from 1.56 – 6.25 µg/mL across the four species tested (*Microsporum canis, Nannizzia gypsea, Trichophyton interdigitale,* and *Trichophyton rubrum)*. This is the first report of phytocannabinoids activity against dermatophytes and aligns with previous observations that unpurified cannabis plant extractions exhibit antifungal activity against dermatophytes [38–40]. New treatment options for the superficial infections caused by dermatophytes, such as tinea known as tinea infections, jock itch, or athlete’s foot, would be welcomed as they are the most common fungal infections, affecting approximately 25% of the general population worldwide [1,41]. Although a handful of promising therapeutic strategies for the treatment of dermatophytosis are in the development pipeline [42–44], cannabinoids may represent a new accessible treatment option that has a simple application, for example by rubbing CBD oils onto topical fungal infections. Our results are promising as an initial observation to establish phytocannabinoids as a treatment for dermatophyte infections and further studies are needed to elucidate the specific pharmacokinetics of CBD and CBDV.

For the remaining moulds, CBD was active against *M. circinelloides* (MIC = 6.25 µg/mL) but not *R. oryzae*, while CBDV was active against *R. oryzae* (MIC = 12.5 µg/mL) but not *M. circinelloides*. Neither CBD nor CBDV had any activity against the five *Aspergillus* species or *F. oxysporum* cpx. The activity observed for at least one phytocannabinoid for *M. circinelloides* and *R. oryzae* is significant as both can cause human fungal infections with high mortality rates, particularly in immunocompromised patients and treatment is often difficult. For example *Mucor* spp. are intrinsically resistant to almost all current antifungal treatments [45] and *Rhizopus* can readily gain resistance [46]. Although further chemical redesign would be ideal to broaden activity and reduce dosage, these are promising initial reports of antifungal activities of phytocannabinoids on clinical-relevant and diverse pathogens.

### Effect of phytocannabinoids on reducing fungal biofilms

The potential of antibiofilm action is important as fungal infections involving biofilms are recognized as a significant clinical problem as they are associated with drug resistance in a wide range of fungi [51–55]. Biofilm formation is essential for *C. neoformans* to survive host immune response and to colonize the central nervous system during infection [56] as well as contribute to drug resistance [57,58]. The ability of fungal pathogens to form biofilms on implanted medical devices is clinically problematic as biofilms enhance the morbidity, mortality and resistance of infections and the use of indwelling medical devices is rapidly growing [57,59–62]. Therefore, we assessed the ability of phytocannabinoids to prevent biofilm formation and disperse mature biofilms for *C. neoformans.*

**Fig 3:**
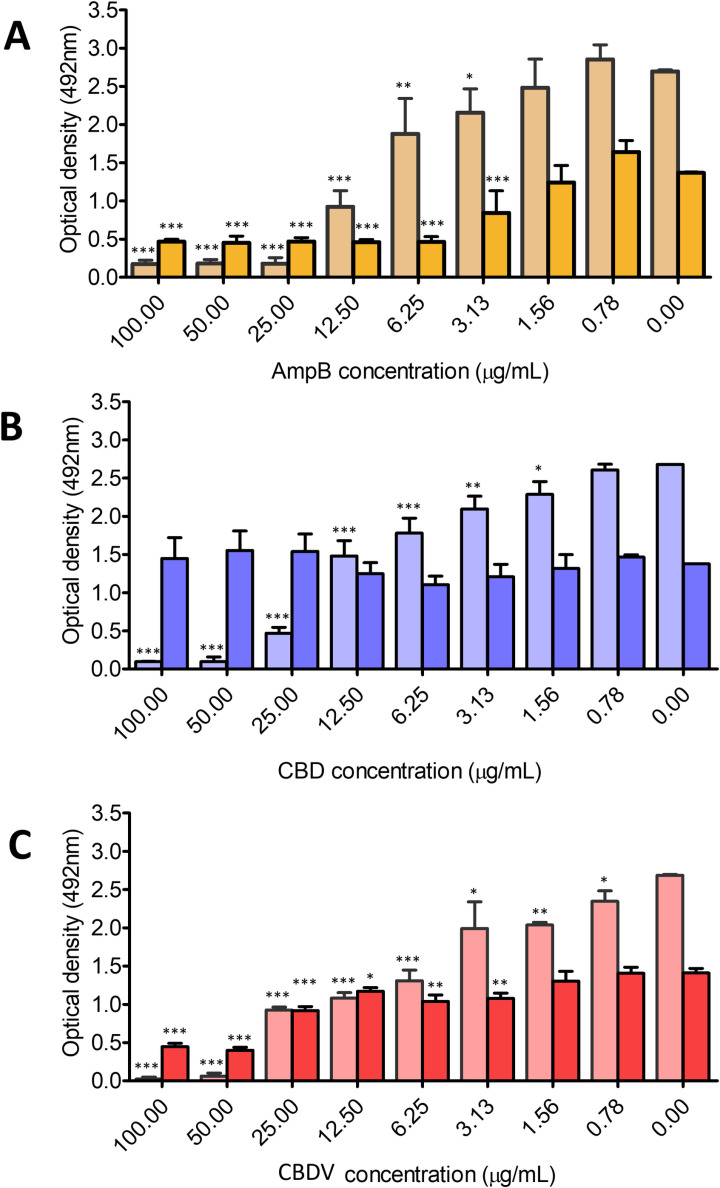
Phytocannabinoids disrupt both developing and mature biofilms of *C. neoformans.* The effect of treatment with various concentrations of (**A**) CBD, (**B**) CBDV, or (**C**) AmpB on *C. neoformans* H99 biofilm formation (shown on the left, in lighter tone) or mature biofilm dissipation (biofilm grown for 48 hr; shown on the right, in darker tone). Metabolic activity was measured by the XTT reduction assay; data presented are the means of three XTT measurements ± SD. Optical density observed from different concentrations of compounds were compared to the control (media only without adding AmpB, CBD, or CBDV) by one-way ANOVA (*p < 0.01, ** p < 0.001, and *** p < 0.0001).

The results showed a significant reduction of our control antifungal AmpB, relative to the untreated control of both biofilm formation and mature biofilms for *C. neoformans* at concentrations starting from 3.13 μg/mL (**Fig 3A**). Promisingly, both CBD and CBDV displayed significantly decreased biofilm formation at even lower starting concentrations of 1.56 μg/mL and 0.78 μg/mL, respectively (**Fig 3B and 3C**), although only CBDV was effective against mature biofilms (at concentrations as low as 3.13 μg/mL; **Fig 3C).** These results provide important insights regarding mechanisms of how the phytocannabinoids are working against *C. neoformans* and ultimately may represent novel strategies for the prevention or eradication of cryptococcal colonization of medical prosthetic devices. This is especially important as biofilms in *C. neoformans* have been linked to increased resistance to existing antifungals, such as amphotericin B and caspofungin, compared to planktonic cells [58] and increased survival within macrophages, complicating both treatment and patient outcomes [56].

### Phytocannabinoid exposure alters *C. neoformans* cell morphology

**Fig 4:**
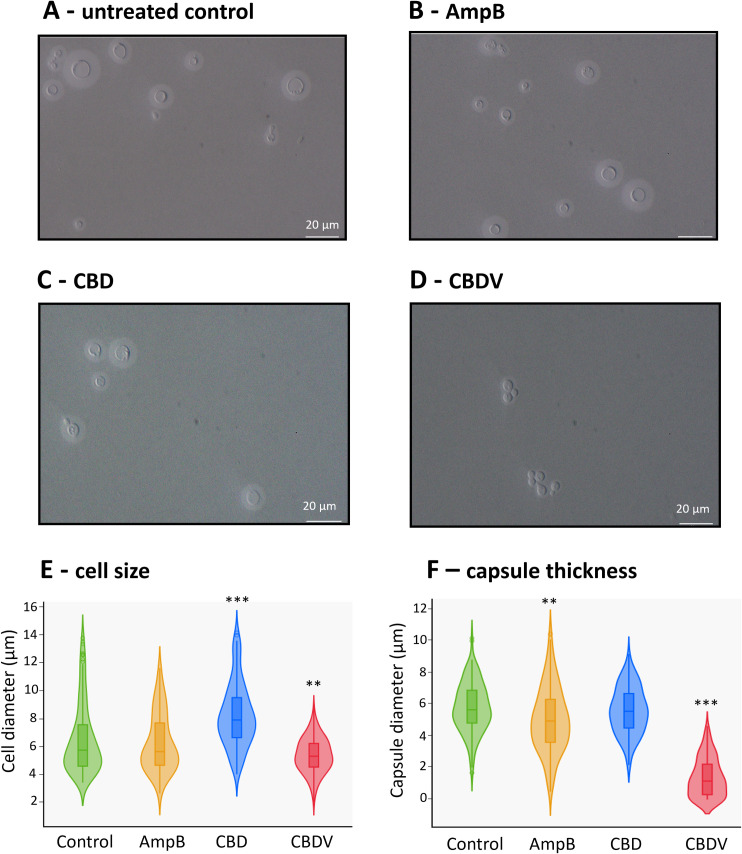
Morphology changes observed in *C. neoformans* under treatment of phytocannabinoids. Cells were counterstained with India ink and captured by light microscopy at 60 × magnification. Representative images of *C. neoformans* cells treated with untreated control **(A)**, AmpB **(B)**, CBD **(C)**, and CBDV **(D)**. Violin plots show the distribution of cell diameter (**E**) and capsule diameter (**F**) across 200 *C. neoformans* cells from two independent biological replicates. Statistical significances were assessed using One-way ANOVA test (**p < 0.01 and ***p < 0.001).

Changes in cell morphology, specifically capsule and cell size, are clinically important for virulence and drug resistance in *C. neoformans* [63,64]. To further investigate the mechanism of action of phytocannabinoids and their impact on changing cell morphology we performed capsule staining and microscopy of *C. neoformans* H99. In our study, AmpB treatment slightly reduced capsule size (5.06 ± 2.09 μm) and did not affect cell size, consistent with previous observations [65]. In contrast, CBD treatment increased cell size but had no impact on capsule size (**Fig 4E and 4F**). CBDV treatment uniquely caused the cells to clump together in bunches (**Fig 4A–D**). Also, cell size and capsule size were significantly reduced following CBDV treatment (cell size: 5.45 ± 1.33 μm; capsule size: 1.52 ± 1.42 μm) compared to the untreated control (cell size: 7.15 ± 3.46 μm, capsule size: 5.88 ± 1.69 μm; **Fig 4E and 4F**). This observed reduction in capsule thickness cannot be attributed solely to the decrease in cell size, as cell size was reduced by a factor of 1.3 with CBDV treatment, while capsule thickness was reduced by a factor of 3.9. To mechanistically assess impacts of phytocannabinoids on metabolic pathways, comparative proteomics analysis was performed.

### Differential proteomics analysis of *C. neoformans* in response to phytocannabinoids

**Fig 5:**
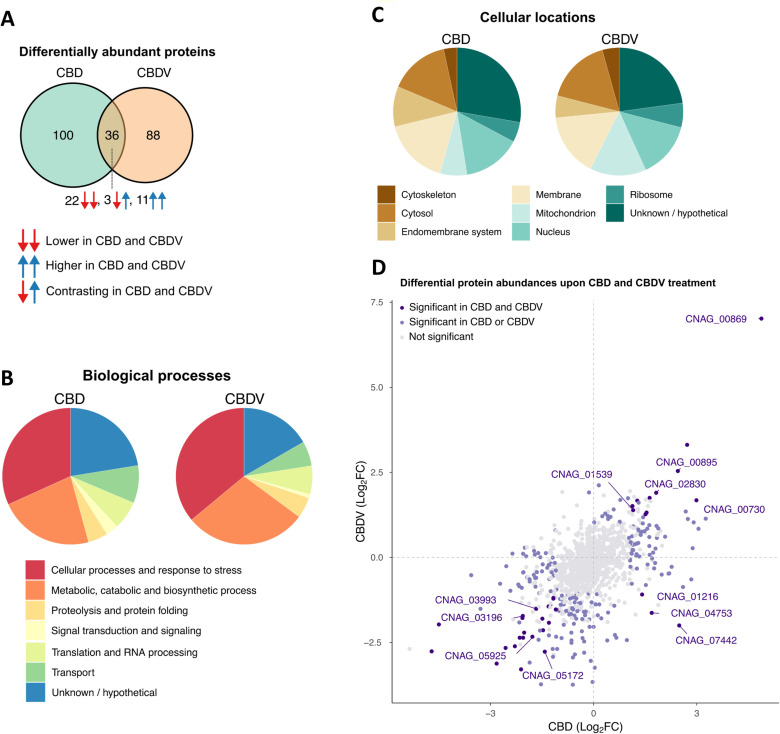
Phytocannabinoid treatment alters the proteome profile of *C. neoformans.* (**A**) Venn diagram showing the number of proteins that were differentially abundant in response to CBD and CBDV treatment (as defined by log_2_FC > 1 and p-value < 0.05 cutoffs) compared to untreated samples. (**B**) Gene ontology (GO) biological process terms (shown on the bottom) assigned to differentially abundant proteins in either CBD or CBDV. (**C**) GO cellular component terms assigned to differentially abundant proteins in either CBD or CBDV. (**D**) Comparison of the log_2_FC values for proteins in response to CBD and CBDV treatment versus the untreated samples. Differentially abundant proteins in response to CBD or CBDV treatment are highlighted in light purple. Differentially abundant proteins in response to both CBD and CBDV treatment are highlighted in dark purple. Representative proteins involved in pathways/processes in response to CBD and CBDV are indicated by their protein IDs.

To further probe the potential fungistatic mechanisms of phytocannabinoids on *C. neoformans*, we performed comparative proteomics to identify the differential abundance changes of proteins after triplicate treatments with subinhibitory concentrations of CBD and CBDV compared to an untreated control. We analysed samples using quantitative mass spectrometry and identified 3,074 proteins representing 41.3% of the 7,429 predicted proteins in *C. neoformans* H99. Of these, 1,117 proteins were quantified by LFQ. When compared to untreated samples, we found 136 and 124 proteins were differentially abundant (as defined by log_2_FC > 1 and p-value < 0.05 cut-offs) in response to CBD and CBDV treatment, respectively (**Fig 5A and Tables A and B in** S1 Table). Gene Ontology (GO) enrichment, performed to uncover gene function, revealed that the number of proteins assigned to biological processes or cellular location were largely conserved, with over half of the proteins for both CBD and CBDV treatment belonging to only two biological classes “Cellular processes and stress response” and “Metabolic, catabolic and biosynthetic processes” (**Fig 5B and 5C and Tables C and D in** S1 Table). Further analysis of the subcellular location of these differentially abundant proteins revealed that for both CBD and CBDV, the most common location was in the membrane (33% and 35% for CBD and CBDV, respectively). Together, this suggests that the membrane and its biogenesis/stability are the most impacted cellular process in fungi after exposure to phytocannabinoids. This align with previous findings that identified membrane disruption as the primary mode of action for cannabidiol acting against Gram-positive bacteria [32,66,67].

We noted that the differences in the location of proteins with altered abundance between CBD and CBDV were largely assigned to the mitochondrion and the membrane (**Fig 5C**). In fact, CBDV displayed double the number of mitochondrial proteins with altered abundance (n = 23 vs 12) and an average log_2_FC of -1.3 compared to CBD which had an average abundance of -0.15 (**Table E in** S1 Table). Removal or reduction of mitochondrial genes has been observed as an effective tolerance strategy for fungi against other antifungal drugs, as it can rearrange lipid and sterol concentrations [68,69]. This difference in fungal cellular response after exposure with the two phytocannabinoids could partially explain some of the differences in antifungal potency and spectrum that we observed (**Table 1**) despite their similar structure (S1 Fig).

Delving deeper into the specific differentially abundant proteins impacted by CBD and CBDV, we observed only ~1/3 (n = 36) were specially shared by both CBD and CBDV treated samples compared to the untreated samples (**Fig 5A**). Among these proteins, we identified numerous proteins essential for survival, virulence, and drug tolerance in *C. neoformans*. Notable proteins affected include those of pyrimidine synthesis pathway, ergosterol biosynthesis pathway, pentose phosphate pathway, inositol pathway, as well as membrane and mitochondrial proteins (**Table 3**). Among these, certain proteins have previously been linked to antifungal tolerance; for instance, myosin-1 (*CNAG_05172*), ABC multidrug transporters AFR1 (*CNAG_00730*) and AFR2 (*CNAG_00869*), and delta(24(24(1)))-sterol reductase (*CNAG_02830*; **listed in Table 3**).

**Table 3:** Differently abundant proteins in response to both CBD and CBDV.

ID	Protein name	CBD		CBDV		Possible function
		Log_2_FC	p-value	Log_2_FC	p-value	
**Proteins that might be important for *C. neoformans* in response to both phytocannabinoids (CBD and CBDV)**						
*CNAG_03196*	Orotate phosphoribosyltransferase	-2.06	2.98E-04	-1.72	1.00E-03	Key enzyme in pyrimidine synthesis pathway that is important for *C. neoformans* high temperature growth and virulence [70]
*CNAG_05925*	Septin ring protein	-1.78	4.91E-03	-2.33	9.59E-04	Essential for proliferation of *C. neoformans* at human body temperature and for virulence [71].
*CNAG_03993*	RP/EB family microtubule-associated protein	-1.67	9.15E-03	-1.51	1.54E-02	Essential for growth, differentiation, and virulence of *C. neoformans* [72]
*CNAG_05172*	Myosin-1	-1.42	2.18E-02	-2.77	5.05E-04	Myosin-1 mutants with altered susceptibility to multiple antifungal drugs in *C. gattii* [73]
*CNAG_01446*	Heat shock protein 9/12	1.27	8.69E-03	1.67	1.85E-03	Stress response [74]
*CNAG_05540*	Urease	1.51	5.52E-03	1.27	1.36E-02	Important virulent factor for central nervous system invasion [75,76]
*CNAG_04014*	26S proteasome regulatory subunit N9	-1.49	3.17E-03	-1.80	9.91E-04	Stress response [77]
*CNAG_06402*	26S proteasome complex subunit SEM1	1.54	7.72E-03	1.32	1.63E-02	
*CNAG_00730*	ABC multidrug transporter AFR1	1.63	2.72E-03	1.75	1.81E-03	Plays important role in pumping triazoles out of the cells, thereby causing antifungal drug resistance [78,79]
*CNAG_00869*	ABC multidrug transporter AFR2	4.88	3.23E-06	7.02	1.73E-07	
*CNAG_02830*	Delta(24(24(1)))-sterol reductase	1.82	2.41E-02	1.90	1.97E-02	Involved in ergosterol biosynthesis pathway. Variants lacking this protein had previously been shown to be sensitive to caspofungin [80]
*CNAG_00895*	Solute carrier family 39 (Zinc transporter), member 1/2/3	2.45	8.56E-03	2.54	7.01E-03	Zinc ion transporter and virulence [81]
*CNAG_04652*	Enoyl reductase	2.72	9.24E-03	3.31	3.13E-03	Fatty acid synthesis [82]
*CNAG_01216*	6-phosphogluconolactonase	1.41	7.98E-03	-1.09	2.63E-02	Involved in the pentose phosphate pathway, which is important for precursor generation of nucleotide and amino acid biosynthesis [83]
*CNAG_04753*	Gluconolactonase	1.69	2.71E-02	-1.63	3.12E-02	
*CNAG_01539*	Inositol-3-phosphate synthase	1.15	6.86E-03	1.39	2.29E-03	Involved in the inositol pathway which is known to function in capsule regulation [84]
*CNAG_07442*	Phosphatidylglycerol/phosphatidylinositol transfer protein	2.49	1.04E-02	-2.00	2.88E-02	
*CNAG_02952*	COX assembly mitochondrial protein	-1.30	4.34E-02	-1.92	7.57E-03	Oxidative phosphorylation
*CNAG_00233*	Mitochondrial import inner membrane translocase subunit TIM14	-1.26	4.28E-02	-1.40	2.83E-02	Mitochondrial protein import
**Other proteins**						
*CNAG_02754*	40S ribosomal protein S12	-4.71	8.56E-05	-2.76	2.99E-03	Structural constituent of ribosome
*CNAG_03779*	Large ribosomal subunit protein uL4m	-2.82	5.87E-04	-3.12	2.93E-04	
*CNAG_00232*	Large subunit ribosomal protein L30e	-2.11	2.54E-03	-3.29	1.38E-04	
*CNAG_04132*	Pre-mRNA-splicing factor CWC2	-2.56	1.49E-02	-2.66	1.25E-02	Removal of introns from mRNA
*CNAG_03944*	Chaperone regulator	-4.50	2.59E-06	-1.97	1.06E-03	Protein folding
*CNAG_02367*	GTP-binding protein ypt3	-2.29	1.56E-03	-2.61	6.80E-04	Protein trafficking
*CNAG_01981*	Sulfide:quinone oxidoreductase	-1.17	8.32E-03	-1.19	7.78E-03	Integral membrane protein
*CNAG_02440*	Cation-transporting ATPase	-1.10	4.46E-03	-1.53	5.88E-04	Membrane transport protein
*CNAG_06168*	S-(hydroxymethyl)glutathione dehydrogenase	-2.05	3.05E-02	-2.36	1.63E-02	Might be involved in conidiation and virulence
*CNAG_01130*	C3H1-type domain-containing protein	-2.02	9.97E-03	-2.21	6.34E-03	Degradation of nuclear RNAs
*CNAG_02814*	Glycerol-3-phosphate dehydrogenase	-1.47	1.92E-02	-2.14	2.68E-03	Carbohydrate metabolism and lipid metabolism
*CNAG_05084*	Phospholipase A-2-activating protein	-1.31	3.02E-02	-1.44	1.98E-02	Necrosis or apoptosis
*CNAG_01850*	histidine kinase	-1.17	4.61E-02	-1.21	4.13E-02	Signal transduction across the cellular membrane
*CNAG_06640*	Carboxypeptidase	2.99	1.13E-03	1.68	2.53E-02	Removing C-terminal amino acids from proteins and peptides
*CNAG_03945*	Uncharacterized protein	-2.15	4.77E-02	-2.36	3.33E-02	Unknown
*CNAG_06511*	Uncharacterized protein	1.13	4.10E-02	1.51	1.18E-02	
*CNAG_05647*	Uncharacterized protein	-2.07	7.94E-04	-1.78	2.06E-03	

^1^ Blue and colouring indicates an increase in protein abundance compared to untreated control, red decreased abundance compared to control.

The proteomic data also reveal some insights into morphology change, specifically capsule loss that was uniquely observed in CBDV treatment. We noticed that one of enzymes in the inositol pathway, phosphatidylglycerol/phosphatidylinositol transfer protein (*CNAG_07442*), showed contrasting abundance levels between CBD and CBDV treatment (**Table 3**). As the inositol pathway plays a crucial role in capsule regulation [83], this may explain the differential capsule loss observed in CBDV but not CBD treatment. Moreover, a previous study reported that *C. neoformans* with a mutation in *man1*, which encodes mannose-6-phosphate isomerase of the GDP-mannose pathway, resulted in smaller, clumping cells and poor capsule formation [84]. In our proteomic data, two proteins in this pathway showed reduced abundance uniquely under CBDV treatment. One of these is mannose-6-phosphate isomerase (*CNAG_04312*), with a log_2_FC of -1.7, consistent with prior findings, and the other is mannose-1-phosphate guanylyltransferase (*CNAG_01813*), with a log_2_FC of -2.69 (**Table B in** S1 Table). This observation suggests that the inositol pathway and GDP-mannose pathway may play important roles in regulating capsule formation in *C. neoformans* in response to phytocannabinoid treatments.

### CBD clearance of *C. neoformans* infection using an *in vivo* wound model

After establishing that the phytocannabinoids CBD and CBDV have broad antifungal properties on a number of clinically-relevant pathogens that appear to act by disrupting biofilm and altering cell morphology via various pathways relating to membrane and metabolism, we assessed the potential efficacy for CBD, the most clinically available phytocannabinoid, to clear a fungal infection *in vivo*.

As a proof-of-concept *in vivo* study, we assessed the topical efficacy of CBD in the insect model *Galleria mellonella*, which has already proven a valuable model for assessing antifungal efficacy [85–88]. While systemic infection via therapeutic injection is the most common route for testing drug efficacy in this model [89], CBD administration via injection is limited due to high serum protein binding (up to >94%), significantly reducing its bioavailability *in vivo* [90]. Thus we opted for the alternative route of topical application of CBD using infection with our model fungal pathogen *C. neoformans*, which is also capable of topical infection [91]. For this, we employed the recently developed *G. mellonella* burn wound model [92,93], which provides an ethical platform for evaluating the effectiveness of topical treatments on fungal wound infections.

**Fig 6:**
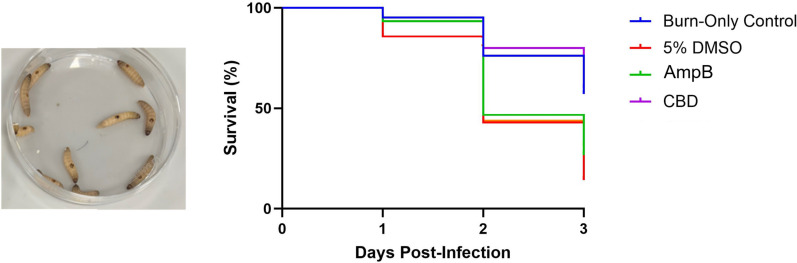
*G. mellonella* burn wound model for testing CBD efficacy against *C. neoformans* infections. The Kaplan-Meier survival curves were plotted based on survival of 15 larvae during 3 days after treatment with either 5% DMSO (vehicle control), AmpB or CBD. Larvae with only burn wound (Burn-Only control) were included to monitor the effect of the burn wound alone. The photo in the left shows the appearance of the Galleria larvae after the wound is introduced.

We tested duplicate groups (7–8 larvae per group), and 1 hour after infection of *C. neoformans* on the wound site, 10µl of CBD 2mg/mL was applied and Galleria were assessed for health and survival over a period of 3 days. We observed a stark, significant increase in the survival of larvae treated with CBD compared to those treated without CBD (5% DMSO; **Fig 6**; Log rank test, p = 0.01). In fact, the effect of *C. neoformans* infection treated with CBD brought the survival rates to near that of the uninfected, burn only control, and appeared to work more effectively than the AmpB control (**Fig 6**). Overall, this pilot study provides compelling preliminary evidence that CBD could be easily adapted for use to treat topical fungal infections in the clinic.

CBD and CBDV, despite having only minor structural differences (**Fig 1**), exhibit distinct variations in their *in vitro* activity, particularly in biofilm disruption, cell morphology, and proteomic profiles. Future research should focus on assessing the *in vivo* efficacy of both CBD and CBDV, as well as studying the pharmacokinetics (PK) and pharmacodynamics (PD) of these phytocannabinoids in higher animal models.

## Conclusion

Overall, this study highlights promising antifungal properties of the phytocannabinoids CBD and CBDV against select fungal pathogens. We demonstrated not only their fungicidal activity against *C. neoformans*, but also their potential effectiveness against wider *Cryptococcus* strains, various other yeasts, and moulds including common dermatophytes, emphasizing their potential broader applicability in the clinic and the community. We demonstrated that the phytocannabinoids appear to work via disrupting biofilms and altering cell morphology, while clear impacts on metabolism and membrane production could be observed with comparative proteomics. We finally showed that for the commonly available CBD, *in vivo* survival of *G. mellonella* was significantly boosted after *C. neoformans* infection, emphasizing the clear potential of CBD as an antifungal. Taken together, the demonstrated efficacy of CBD and CBDV as broad antifungal agents, coupled with their established safety profile, makes them an exciting resource as a foundation for the development of future therapeutic interventions.

## Materials and methods

### Fungal strains and reagents

*C. neoformans* var. *grubii* strain H99 was used throughout the study, unless specifically stated. Strains were stored in 15% glycerol at −80 °C until use, at which point they were grown on solid YPD (2% bacteriological peptone, 1% yeast extract, 2% glucose, 2% agar) for 2 d at 30 °C. Cultures were freshly made at each time of use. Liquid culture was made by culturing *C. neoformans* overnight (~16–18 hr) in YPD broth at 30 °C with shaking at 180 rpm.

The (-) isomers of two phytocannabinoids were used in this study are cannabidiol (CBD) and cannabidivarin (CBDV). These phytocannabinoids were obtained from the National Measurement Institute (North Ryde, NSW Australia) and LipoMed AG, (Arlesheim, Switzerland). Phytocannabinoids were dissolved in DMSO to the concentration of 10 mg/mL. AmpB was used as an antifungal control (Sigma-Aldrich, USA).

### Minimum inhibitory (MIC) assays

#### C. neoformans.

The MIC for each compound against *C. neoformans* H99 was determined via the broth microdilution method according to Clinical and Laboratory Standards Institute (CLSI) guidelines for fungal susceptibility testing with minor modifications (CLSI M60 - 2017, and CLSI M27 - 2017). Briefly, a single colony from a YPD plate was picked and emulsified in 1 mL of sterile PBS. Cells were counted using a hemocytometer, and the concentration of the cell suspension was adjusted to 2–5 × 10^6^ cells/mL. The cell suspension was diluted 1:1000 in RPMI-1640 medium (pH 7.4 or pH 4.0) or YNB to obtain the 2 × stock suspension for the MIC assay. A two-fold dilution series of 100 µL containing either CBD or CBDV in supplemented RPMI-1640 medium (pH 7.4 or pH 4.0) was added into 96-well flat-bottom microplates (Garnier, polystyrene; final concentration ranging from 0.78 to 25 µg/mL), followed by the addition of 100 µL of cell culture (final cell concentration of ~3 × 10^3^ cells/mL). After incubation at 37 °C for 48 hr in a humidified chamber, cells were resuspended in each well and absorbance was measured at 405 nm with a microtiter plate reader (SpectraMAX 190, Molecular Devices). AmpB were used as antifungal controls. For all conditions, the 100% MIC was determined, as compared to the untreated control. To determine the minimum fungicidal concentration (MFC), 200 µL from each well showing no visible growth was plated on YPD after resuspension and incubated for 48 hr at 30 °C. Two independent biological replicates were carried out for each condition.

#### Other fungal species.

Thirty-three fungal strains were screened encompassing clinical, veterinary, and environmental isolates. The strain name and source of each isolate is detailed in **Table 2**. Yeast strains and non-dermatophyte moulds were maintained as glycerol stocks at -80 °C and grown on potato dextrose agar (PDA; Oxoid) for 24–48 h before use. Dermatophytes were maintained as agar cuts in water and grown on PDA between 48 hr and seven days until sporulation was obtained. MIC assays were performed as described above for *C. neoformans* with minor modifications. For yeasts, single colonies were picked from PDA plates, suspended in PBS, and adjusted to a final concentration of 0.5-2.5 × 10^3^ cells/mL. For moulds, spore suspensions were produced by gently agitating sporulating colonies in PBS with a drop of Tween 20 detergent and adjusted to a final concentration of 0.4-5 × 10^4^ spores/mL for non-dermatophyte moulds and 1–3 × 10^3^ spores/mL for dermatophytes. All tests used RPMI-1640 (Sigma-Aldrich, USA) supplemented with 0.165 M MOPS and 2% D-glucose (pH 7). Antifungal agents were assayed at concentration ranges of 0.0039 to 25 µg/mL for CBD and CBDV and 0.03125 to 4 µg/mL for AmpB. Plates were incubated without agitation for 35 °C for 24 hr (*Candida, Aspergillus, Mucor, Rhizopus, Fusarium*), 48 hr (*Cryptococcus*), or 144 hr (dermatophytes). The MIC was determined visually and defined as the lowest drug concentration at which growth was completely inhibited.

### Time-kill assays

An inoculum of 10^6^ cells of *C. neoformans* H99 was aliquoted into 10 mL of YPD broth containing either CBD, CBDV, or AmpB at their respective MIC and ½ MIC. Cultures were incubated at 30 °C with shaking at 200 rpm. At 0.5, 1, 2, 4, and 24 hr post-inoculation, 100 µL aliquots were withdrawn, serially diluted in PBS and back-plated onto YPD. Colonies were counted after 48 hr of incubation at 30 °C to determine viable cell density. Three technical replicate plates were counted for each experiment, and two biological replicates were performed.

### Biofilm inhibition assay

#### Effect of phytocannabinoids on *C. neoformans* biofilm formation.

The susceptibility of *C. neoformans* biofilms to phytocannabinoids was examined as previously described [58] with some modifications. Briefly, a two-fold dilution series of the 100 µL solution containing either CBD or CBDV in RPMI-1640 medium (pH 7.4) was added into 96 well flat-bottom microplates (Garnier, polystyrene; final concentration between 0.78 and 100 µg/mL), followed by the addition of 100 µL of inoculum (final cell concentration of 10^7^ cells/mL). AmpB was also included as a positive control at a final concentration between 0.016 and 2 µg/mL. Three wells with only RPMI-1640 medium was included as a negative control. The plate was incubated at 37 °C for 24 hr. After 24 hr of incubation, the wells were washed three times with 0.05% Tween 20 in Tris-buffered saline to remove non-adherent cells and biofilm formation was quantified by the XTT reduction assay.

#### Effect of phytocannabinoids on *C. neoformans* mature biofilm.

The inhibition of mature biofilms produced by *C. neoformans* was investigated using the XTT reduction assay [58]. Overnight broth cultures of each strain were counted and adjusted to 1 × 10^6^ cells/mL in RPMI-1640, then 100 µL of the cell suspension was transferred into 96-well microtiter plates. Plates were then incubated for 48 hr at 37 °C, the media aspirated, and mature biofilms washed three times with PBS to remove non-adherent planktonic yeasts. Serial two-fold dilutions starting at 100 µg/mL solutions were prepared for CBD, CBDV, or AmpB in RPMI-1640, and 200 µL of each concentration was added to biofilms. After a further 48 hr of incubation at 37 °C, wells were washed three times with 0.05% Tween 20 in Tris-buffered saline to remove non-adherent cells and mature biofilm was quantified by the XTT reduction assay.

### XTT reduction assay

To determine the metabolic activity of yeast cells within the biofilm, 50 µL of XTT salt solution (1 mg/mL in PBS) and 4 µL of menadione solution (1 mM in acetone; Sigma Chemical Co.) were added to each well of the 96 well plate. The microtiter plates were incubated at 37 °C for 5 hr in the dark. Metabolic activity was measured via mitochondrial dehydrogenase activity, which reduces XTT tetrazolium salt to XTT formazan, resulting in a colorimetric change, which was measured with a microtiter reader (PHERAstar- BMG Labtech) at 492 nm.

### India ink staining to measure capsule and cell size

Capsule induction was performed as previously described [94] with minor modifications. Briefly, *C. neoformans* strain H99 was grown (from a single colony on YPD media) overnight for 16 hr at 30 °C with 200 rpm shaking in YPD media. Overnight cultures of *C. neoformans* were washed twice at 7,000 × *g* for 1 min in sterile PBS and resuspended in PBS. Total cell concentration was quantified using a hemocytometer and adjusted to a final cell count of 1.25 × 10^5^ cells/mL in 5 mL of RPMI-1640 with 0.165 M MOPS (pH 7.4). Cells were incubated in 6-well plates, untreated, or in the presence of sub-inhibitory concentrations that was used for proteomic experiment [CBD (3.125µg/mL), CBDV (6.25 µg/mL), or AmpB (0.25µg/mL)] at 37 °C with 5% CO_2_ for 72 hr without shaking. Samples were prepared for microscopy by harvesting cells at 7,000 × *g* for 1 min and resuspending the cell pellet in 150 μL of PBS and 10 μL India ink (Winsor & Newton). Counterstained yeast cells were observed microscopically, and images were captured with an Olympus BX53 light microscope equipped with a DP28 digital camera set to 60 × magnification. Images were acquired using CellSens software (Evident). Cell size and capsule diameter were measured using FIJI software [95]. Capsule diameter was defined as the difference in distance of the volume of the whole cell (yeast cell inclusive of capsule) and volume of the cell body (no capsule). A total of *n* = 200 cells were measured per condition across two biological replicates.

### Whole cell preparation for proteomics

To determine whole cell proteomic changes that occur during exposure of low-level phytocannabinoids, protein purification was performed based on previous methodologies [96,97]. For this, a single colony of *C. neoformans* H99 was inoculated into 5 mL of RPMI-1640 liquid medium and incubated overnight at 30 °C with shaking at 180 rpm. This overnight culture was used to inoculate 10 mL of RPMI-1640 liquid medium to a starting OD of 0.01. Triplicate cultures were supplemented with either ½ MIC CBD (3.13 µg/mL), CBDV (6.25 µg/mL), or unsupplemented as a control and grown for 16 hr at 30 °C with shaking at 180 rpm. Cells were harvested upon reaching an OD600 nm of 1.0 and collected by centrifugation at 2500 × *g* for 10 min at 4 °C and washed with 10 mL of ice-cold lysis buffer (100 mM Tris HCl buffer pH 8.0, 0.1 mM EDTA, 1 mM phenylmethylsulfonyl fluoride, 1 x complete protease inhibitor cocktail). After washing, cells were resuspended in 400 μL of ice-cold lysis buffer and subjected to mechanical bead-beating with 0.5 mm glass beads, using the Cryolys Evolution homogenizer (Bertin Technologies, France), for 8 cycles of 1 min at 4 °C, followed by a 2 min rest. Sample tubes were then perforated at their base and placed into 15 mL falcon tubes (Sarstedt, Germany) and centrifuged at 500 × *g* for 2 min. Beads were washed and centrifuged again with an additional 600 μL of ice-cold lysis buffer. Cell lysates were transferred to Protein LoBind tubes (Eppendorf, Germany) and cell debris was removed from the lysate by centrifugation at 2,000 × *g* for 10 min at 4 °C. Sample lysates were then denatured by addition of SDS (Invitrogen, USA) to a final concentration of 0.5% and DTT (Bio-Rad, USA) to a final concentration of 10 mM, and incubated at 60 °C for 30 min. After cooling to room temperature, cysteines were alkylated by addition of iodoacetamide (Bio-Rad, USA) to a final concentration of 50 mM and incubated at room temperature for 1 hr. Protein was precipitated by addition of 4 volumes 1:1 methanol:acetone and incubated at −20 °C for 16 hr, harvested via centrifugation at 18, 000 × *g* for 10 min, resuspended in 100 mM Ammonium bicarbonate (Sigma-Aldrich, USA), and digested with trypsin overnight at 37 °C with shaking (Promega, USA). Peptides were desalted using Bond Elut Omix tips (Agilent Technologies, USA).

### Nanoflow liquid chromatography – tandem mass spectrometry

Extracted peptides were analysed on an Exploris 480 mass spectrometer (Thermo, San Jose) connected to a Vanquish Neo nanoLC system (Thermo, San Jose, CA, USA). Chromatographic separation was performed on a reversed-phase fused silica capillary column, 75 μm I.D. x 15 cm, packed with Dr Maisch Reprosil Pur C18 AQ, 120 Å, 3 μm packing material, at 45 °C. Before the analytical column, peptides were loaded on a trap column 300 μm × 5 mm (Thermo Acclaim PepMap C18 100) [98]. Samples were loaded in 0.1% v/v FA, and peptides were resolved over a 60 min gradient with a flow rate of 300 L/min. The 60 min gradient was developed using buffer A (0.1% v/v formic acid) and buffer B (80% v/v ACN, 0.1% v/v FA) as follows: 2% buffer A (2 min), 40% buffer B (60 min), 80% buffer B (7 min). The spectral acquisition was performed in positive ion mode over a scan range of 400 m/z to 1500 m/z (60,000 resolution, 25 ms maximum injection time). Data-dependent acquisition method was used to acquire MS/MS spectra over a 1.5 second cycle time (15, 000 resolution, 50 ms maximum injection time, 1.2 m/z isolation width, dynamic exclusion enabled for 20 s).

### Proteomic data analysis

Peptide-to-spectrum matching (PSM) of the Thermo raw files was performed using MSFragger (version 3.8) via FragPipe (version 20) [99,100]. A closed search was performed against the *C. neoformans* proteome database (7,813 annotated proteins; project PRJNA411) [101] with decoy and contaminant protein sequences automatically generated by Fragpipe. Enzyme specificity was set to trypsin with an allowance for up to one missed cleavage. Peptide search parameters were set to a sequence length between 7 and 50 amino acids and a mass range of between 300 and 5000 Da with a ± 20 ppm mass tolerance at the precursor and fragment ion level. Oxidation of methionine and N-terminal acetylation were set as variable modifications while carbamidomethylation of cysteine was a fixed modification with an allowance for up to five modifications per peptide. PeptideProphet and ProteinProphet in Philosopher (version 5.0.0) [102,103] were used to ensure a maximum protein and PSM false discovery rate (FDR) of 1%. Decoy and contaminant identifications were removed automatically by FragPipe. Label free quantitation (LFQ) was performed using IonQuant 1.9.8 [104] with the following parameters: LFQ set to MaxLFQ using a minimum of two ions; match between runs with a retention time tolerance of 1 min and 10 top runs; normalise intensity across runs; and peptide-protein uniqueness set to “unique + razor”. Peak tracing was set to require a minimum of three scans with a ppm tolerance of 10 m/z and a retention time tolerance of 0.4 min.

Results from FragPipe were uploaded to FragPipe Analyst (fragpipe-analyst.nesvilab.org/) for analysis and visualization with the following parameters: Intensity type was MaxLFQ Intensity; minimum percentage of global missing values was set to 50%; minimum percentage of missing values in at least one condition was set to 50%; p-value cut-off of <0.05; log_2_FC cut-off of 1; normalization was performed using variance stabilizing normalization; imputation using Maximum Likelihood Estimation (MLE). Gene Ontology (GO) enrichment analysis was performed with the topGO R package [105] using gene-to-GO mappings from Uniprot, while KEGG pathway enrichment analysis was performed with the clusterProfiler [106] R package. All p-values were adjusted using Benjamini-Hochberg p-value correction. GO terms and KEGG pathways with adjusted p-value < 0.05 were considered significant. Specific biological pathways impacted by CBD and CBDV treatment were also identified by using KEGG Mapper to map genes to *C. neoformans var. grubii* H99 pathways in KEGG [107]

### *Galleria mellonella in vivo* model

The *G. mellonella* burn-wound model was conducted as described previously [92,93]. Briefly, *G. mellonella* larvae of weight above 200 mg were sterilised by spraying with 80% ethanol, which was then allowed to evaporate by leaving petri dishes open. A steel nail, with head of 1.8 mm width attached to cork was used to administer burns. Head of the nail was placed in Bunsen flame until red-hot, removed from the heat and cooled for 4 s, then immediately placed onto the middle segment of the larva back and held for 4 s. Any larvae that displayed excessive haemolymph leakage or fat body protrusion were immediately placed at -20 °C to euthanise. Twenty minutes after burn administration, 2 µL of *C. neoformans* H99 at concentration of 1x10^10^ cells/mL was pipetted onto the wound. Larvae were allowed to rest for 1 hr before pipetting 10 µL of treatment onto the wound. Treatments included 5% DMSO (vehicle control), AmpB 0.875 mg/mL (antifungal control), and 2 mg/mL CBD. Larvae with only burn wound were included to monitor the effect of the burn wound alone. All larvae were kept at 37 °C and survival monitored daily for 3 d. Mortality was recorded upon complete loss of motility with external stimulation. Two separate biological replicates (n = 15 larvae in total) were performed.

## Supporting information

S1 Fig Minimum fungicidal concentrations of CBD (a), CBDV (b) and AmpB (c) against *C. neoformans* H99. (TIF)

S2 Fig FIC checkerboard assay results ΣFIC values generated are shown for CBDV/ CBD with existing antifungals (Fluconazole/ AmpB) that was tested against *C. neoformans* H99. The concentrations of CBDV/CBD (µg/mL) are shown on the y axis and concentrations of AmpB/ Fluconazole in the x axis in μg/mL. Concentrations where growth was detected based on OD600 absorbance are filled in red. The combination is considered to interact synergistically at ΣFIC ≤ 0.5, additive interaction at ΣFIC > 0.5 – 1, indifferent interaction at ΣFIC 1–4 and antagonistic interaction (ΣFIC > 4).
(TIF)

S1 Table **A.** Differentially abundant proteins in *C. neoformans* H99 in response to CBD treatment. **B**. Differentially abundant proteins in *C. neoformans* H99 in response to CBDV treatment. **C**. Significantly enriched GO terms based on differentially abundant proteins in CBD treatment. **D**. Significantly enriched GO terms based on differentially abundant proteins in CBDV treatment. **E**. Differentially abundant proteins in both CBD and CBDV treatment classified as mitochondrion.
(XLSX)
