# Supplementary figures and images for: Uncovering the antifungal potential of Cannabidiol and Cannabidivarin
Source: PLoS Negl Trop Dis. 2025 Jun 5;19(6):e0013081. doi: 10.1371/journal.pntd.0013081 (PMC12139526; doi:10.1371/journal.pntd.0013081)

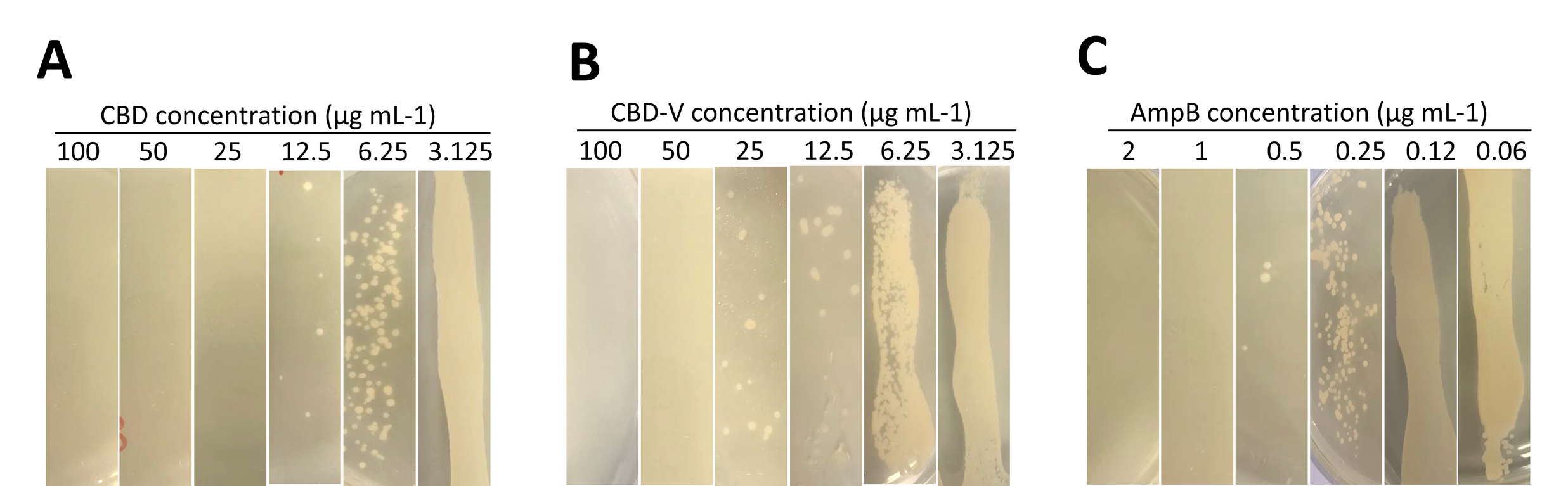

Supplement: S1 Fig — (TIF) [file pntd.0013081.s001.tif]

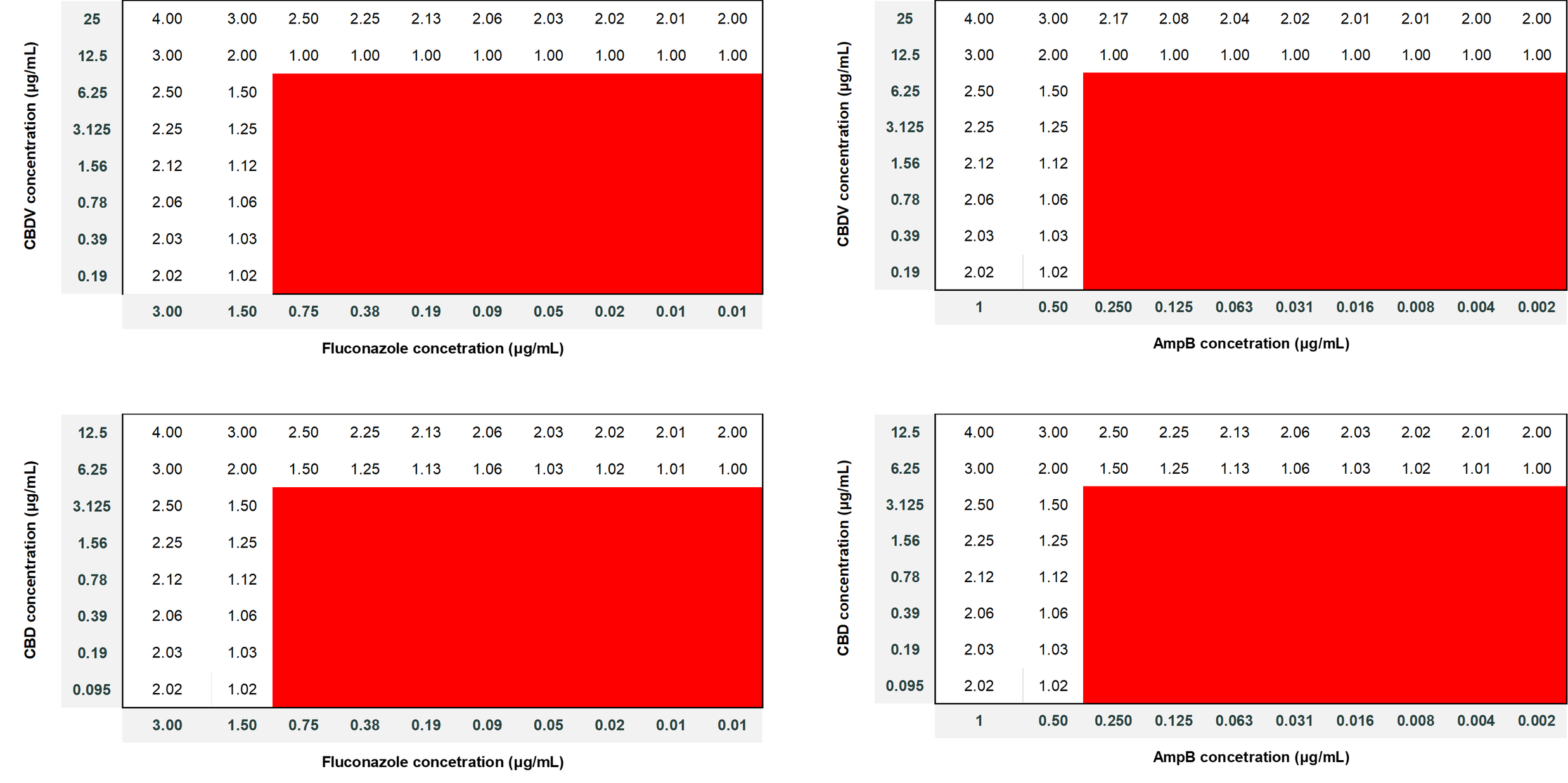

Supplement: S2 Fig — The concentrations of CBDV/CBD (µg/mL) are shown on the y axis and concentrations of AmpB/ Fluconazole in the x axis in μg/mL. Concentrations where growth was detected based on OD600 absorbance are filled in red. The combination is considered to interact synergistically at ΣFIC ≤ 0.5, additive interaction at ΣFIC > 0.5 – 1, indifferent interaction at ΣFIC 1–4 and antagonistic interaction (ΣFIC > 4). (TIF) [file pntd.0013081.s002.tif]
